# Effect of a new motorway on social-spatial patterning of road traffic accidents: A retrospective longitudinal natural experimental study

**DOI:** 10.1371/journal.pone.0184047

**Published:** 2017-09-07

**Authors:** Jonathan R. Olsen, Richard Mitchell, David Ogilvie, on behalf of the M74 study team

**Affiliations:** 1 MRC/CSO Social and Public Health Sciences Unit, Institute of Health and Wellbeing, University of Glasgow, Glasgow, United Kingdom; 2 Centre for Research on Environment, Society and Health (CRESH), Institute of Health and Wellbeing, University of Glasgow, Glasgow, United Kingdom; 3 MRC Epidemiology Unit & UKCRC Centre for Diet and Activity Research (CEDAR), School of Clinical Medicine, University of Cambridge, Cambridge, United Kingdom; TNO, NETHERLANDS

## Abstract

**Background:**

The World Health Organisation reports that road traffic accidents (accidents) could become the seventh leading cause of death globally by 2030. Accidents often occur in spatial clusters and, generally, there are more accidents in less advantaged areas. Infrastructure changes, such as new roads, can affect the locations and magnitude of accident clusters but evidence of impact is lacking. A new 5-mile motorway extension was opened in 2011 in Glasgow, Scotland. Previous research found no impact on the number of accidents but did not consider their spatial location or socio-economic setting. We evaluated impacts on these, both locally and city-wide.

**Methods:**

We used STATS19 data covering the period 2008 to 2014 and describing the location and details of all reported accidents involving a personal injury. Poisson-based continuous scan statistics were used to detect spatial clusters of accidents and any change in these over time. Change in the socio-economic distribution of accident cluster locations during the study period was also assessed.

**Results:**

In each year accidents were strongly clustered, with statistically significant clusters more likely to occur in socio-economically deprived areas. There was no significant shift in the magnitude or location of accident clusters during motorway construction or following opening, either locally or city-wide. There was also no impact on the socio-economic patterning of accident cluster locations.

**Conclusions:**

Although urban infrastructure changes occur constantly, all around the world, this is the first study to evaluate the impact of such changes on road accident clusters. Despite expectations to the contrary from both proponents and opponents of the M74 extension, we found no beneficial or adverse change in the socio-spatial distribution of accidents associated with its construction, opening or operation. Our approach and findings can help inform urban planning internationally.

## Introduction

Road traffic accidents (accidents) have a significant impact on public health and the World Health Organisation reports that, without action, they will become the seventh leading cause of death globally by 2030 [1]. The impact of accidents is socially and spatially unequal; accidents often occur in spatial clusters [2] and, generally, there are more accidents in, and more casualties from, less advantaged areas [3], despite their being less likely to own a car [4, 5]. Spatial clustering of accidents occurs because they are not random events [6]. Accidents are influenced by human, road and environmental factors [7, 8]; these factors are not mutually exclusive and their impact can be individual or combined. The identification of an accident cluster is important because it can prompt the implementation of traffic safety measures or identification of environmental factors which may be linked to an increased or decreased number of accidents. Understanding geographic variation in accidents is also important for accident prevention and to highlight potential causal factors at a small area local level.

Few studies have examined spatial clustering of accidents. A study in Spain, for example, examined the locations of accidents involving wild animals, identifying areas with a higher spatiotemporal risk of accidents to aid future mitigating actions [9]. Methodologically, many existing studies have mapped accident locations using Geographical Information System (GIS) software to visually identify high risk areas [2, 10, 11], but they tend not to have formally identified statistically significant ‘clusters’ or considered their temporal stability [12]. Visual identification of ‘hot spots’ is subjective. The availability of datasets which provide the precise geo-coordinates of accidents has improved dramatically in recent years [11], but the opportunity this provides for more robust assessment of spatial patterns remains relatively unexplored.

The causes of accidents and their locations can change over time due to factors such as new road layouts [13], changes in speed limits [14] and traffic calming measures [15]. It might be assumed that new road layouts would have an impact on accidents, both during their construction and on opening; perhaps as people travel in unfamiliar environments human error might be more likely to result in an accident. Alternatively, perhaps the unfamiliarity of a new road layout or a construction zone would prompt greater caution and reduce the risk of an accident [16]. Where new road design is good, a long term reduction in accidents might be expected, but poor design might generate a new accident cluster. Interventions in road systems and infrastructure ought, therefore, to be carried out with likely impacts on accidents in mind. However, there are very few published examples in which new road infrastructure has been evaluated [17]. Although somewhat dated evidence suggests reductions in accidents following infrastructural changes in Western Europe and North America [18], our recent study evaluating effects of the M74 motorway extension in Glasgow found no evidence of impact on the numbers of accidents, either locally or city-wide [19]. However, focusing on accident counts might have missed other effects, in particular the possibility that accident clusters might have been shifted to other areas of the city, as suggested by some local residents who participated in community and stakeholder engagement events linked to the study [20].

### The intervention

The M74 motorway extension is a 5-mile stretch of new motorway in the south of Glasgow, Scotland which opened in June 2011. The new road cost approximately £800 million, is mainly raised above existing roads and dwellings, and crosses a largely and predominantly urban residential area. An independent local public inquiry in 2003 considered the arguments for and against construction and concluded that the claimed benefits were likely to be ‘ephemeral’ and that the new motorway ‘would be very likely to have very serious undesirable results’ for local communities. It therefore recommended against the proposal [21]. Nevertheless, the construction went ahead. Two of the key strategic and economic objectives for the construction of the motorway extension were to relieve congestion on local streets across the city and to reduce road accidents [22]. It is possible that the M74 extension may have contributed to changing the spatiotemporal distribution of accidents–for example, by shifting the burden of accidents into or out of more deprived neighbourhoods. The main objectives of this study were therefore to describe the socio-spatiotemporal clustering of accidents in Glasgow; and to evaluate whether the construction or opening of the M74 motorway extension shifted the location of accident clusters in the city in general, and into more or less deprived areas of the city in particular.

## Materials and methods

### Design and study area

**Fig 1:**
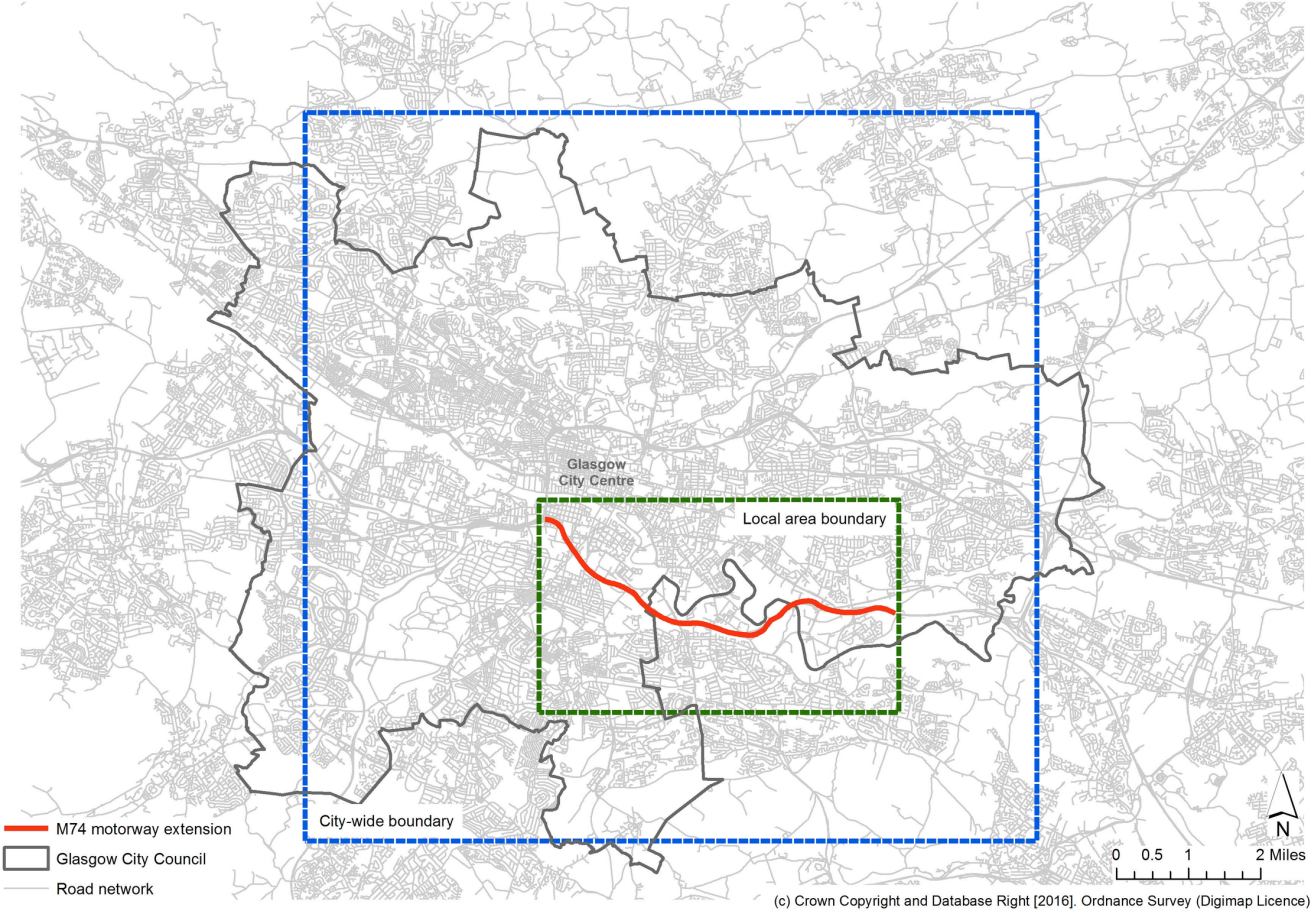
Study area boundaries. Reprinted from Edina Digimap under a CC BY license, with permission from Ordnance Survey, original copyright 2016.

The M74 motorway extension was constructed in the south of Glasgow (Fig 1), but is linked to other major roads in the west of Scotland and the north of England. The extension therefore had potential impacts upon the transport network both locally and city-wide–taking local traffic off minor local roads, but also taking longer-distance traffic, that previously needed to pass through the city, literally overhead. In an evaluation of changes in traffic flow one year after opening, Transport Scotland described ‘significant changes’ across the city; increases were observed on the main arteries leading to the motorway, with decreases on some local streets and other roads in the city [23]. For this reason, we used two different definitions of study area: first, the local area surrounding the M74 motorway extension; and second, a wider area near the perimeter of the Glasgow City Council boundary (both defined as quadrilaterals for the purposes of the spatial analytical procedure) (Fig 1). For each definition of study area, the location, magnitude and temporality of any clusters in accidents was evaluated. Had we observed any significant change associated with the periods of construction, opening or subsequent use of the M74 extension, it would have indicated a possible intervention effect.

### Road traffic accidents

Routinely collected accident data were obtained from the UK Department for Transport STATS19 dataset for the period 2008 to 2014. STATS19 provide precise geo-coordinates of all accidents in the UK which have resulted in a casualty and been reported to the Police. Accidents are classified within the database using the following definitions: *Slight*, an accident in which at least one person is slightly injured but no-one is killed or seriously injured; *Serious*, in which at least one person is seriously injured but no-one is killed; and *Fatal*, in which at least one person is killed [24]. No spatially comprehensive or temporally consistent traffic flow data were available for our study, because traffic counter locations were changed after the opening of the M74 and consequently could not be used to provide a denominator for accidents [19].

### Socio-economic setting

Each accident was linked to the Scottish datazone (a small areal unit used for statistical reporting, containing between 500 and 1,000 household residents) within which the accident occurred. The Scottish Index of Multiple Deprivation (SIMD) score and quintile (relative to the whole of Scotland) of the datazone was then assigned to each accident. The SIMD combines 38 indicators across domains such as income, employment, health, education, skills and training, housing, geographic access and crime [25]. It is a very well established, robust and widely used measure of socio-economic deprivation. The socio-economic position of the individuals involved in the accidents was not available.

### Analysis

Descriptive statistics of accident clusters by year, socio-economic setting and severity classification were assessed prior to spatial analysis.

#### Detection of accident clusters

SaTScan™ is a well-established tool that facilitates a variety of spatiotemporal cluster analyses. These are based on various probability models, and the tool can identify geographically defined clustered areas of high risk, low risk, or both, for the occurrence of an accident compared to the whole geographical area in question [26]. In brief, the software constructs a large number of different sized circular frames with varying location and radii across the study area and then makes a comparison of the occurrence of accidents within each frame with their occurrence outside the frame. The close location of frames with apparently higher rates of accidents is used to identify the location and size of a cluster, and its significance is then determined [27]. For cluster detection, we used a Poisson-based continuous scan statistic to detect spatial clusters of accidents from 2008 to 2014. The model uses a space-time permutation model that is useful when only case or count data are available, as is the case for our dataset [28]. The analysis was performed for two subgroups—all accidents with a slight, serious or fatal casualty, and only those with a serious and fatal casualty—using SaTScan™ v.9.4.2 (http://www.satscan.org/).

The spatiotemporal cluster analysis approach is used increasingly in real-time surveillance, specifically to identify geographic disparities in the incidence of cases of diseases over time, such as cancer [29], tuberculosis [30] and HIV/AIDS [31]. The New York City Department of Health and Mental Hygiene use the space–time permutation scan statistics to monitor daily for potential outbreaks of 35 reportable diseases [32]. Although used infrequently in examining accident clusters over time, the nature of accidents, in terms of having a precise location, lends itself to this method; particularly in the UK where precise accident locations are now routinely collected by the national government.

For disease surveillance, the procedure can identify small geographical areas with higher than expected number of cases than the whole area under investigation. This analysis is equally important for accident cluster detection, where identifying small geographic areas with a higher than expected numbers of crashes is important for targeted accident prevention. Although the procedure has been used infrequently for accident cluster detection, this may be due the fact that until recently there has been a lack of routinely collected data available. As opposed to its use for traditional disease surveillance where historically incidence data have been well recorded. The spatiotemporal function scans the same geographical area to examine changes in the location of clusters over time, while being able to detect overall longitudinal changes in accident numbers. This is of key importance when examining accident number due to an annual decrease in total accident numbers in the UK.

Analysis was performed year by year using accident data for the period 2008 to 2014. The software provided an output of the centroid of each cluster, its size (radius) and its statistical significance. Significant (p<0.05) clusters detected in SaTScan™ were mapped using ArcGIS (10.2.2) to display their centroids and sizes. We applied a cluster limit of 500 Cartesian units (coordinate system, applying a single unit of length for both axes) for the statistical analysis; it is considered good practice that clusters are made as small as possible to ensure that low-risk neighbourhoods are not incorrectly included in a larger high-risk area as it is possible to sustain statistical significance over a large geographical area which can encompass low-risk areas [33].

#### Changes in the location of accident clusters, over time

The spatial-temporal analysis function of the SaTScan™ software used a Poisson-based spatial temporal scan statistic to detect changes in the locations and size of clusters year to year. The procedure compares change in the number of accidents in a specific location (the search radius varying from zero to the predetermined limit) with change in other areas in the wider study area. For example, if during a particular year a road intersection has an increased number of accidents, but a similar increase in accidents was also observed elsewhere in the wider study window, it will not detect the creation of a new spatial-temporal cluster. The analysis output provided the year, direction, location and statistical significance of any changes.

#### Sensitivity analysis

One limitation of the SaTScan™ software is that it may not identify clusters which are located on, or very close to, study area boundaries [34]. Since the accident dataset was available for the entire UK, it was possible to compare results for slightly different definitions of the study area. We compared three different definitions to check for any influence on results, but found that detection of clusters remained consistent (details of boundary coordinates, S1 Table). Our results were therefore not sensitive to boundary definition.

### Results

#### Annual count of clustered accidents and socioeconomic status

**Fig 2:**
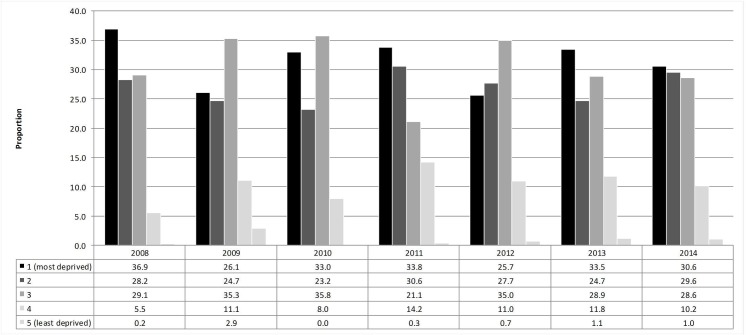
Annual proportion of clustered accidents and area level deprivation quintile of accident location. Graph presents annual proportions of clustered accidents by deprivation quintile for the period 2008 to 2014.

We previously reported that the annual count of accidents in Glasgow declined by 50.6% between 1997 and 2014 [19]. The count declined by 25.1% during the shorter time period under consideration here, from 2008 (n = 3,885) to 2014 (n = 2,908) (S2 Table). The annual proportion of clustered accidents (i.e. those accidents which formed part of identified and statistically significant spatial clusters) decreased by 37.6% from, 2008 (n = 471) to 2014 (n = 294) (S3 Table). Fig 2 shows the distribution of clustered accidents by socio-economic deprivation quintile over time, highlighting that a large majority of accident clusters occurred in the three most deprived quintiles, albeit with some annual fluctuation.

#### Spatial location of accident clusters

**Fig 3:**
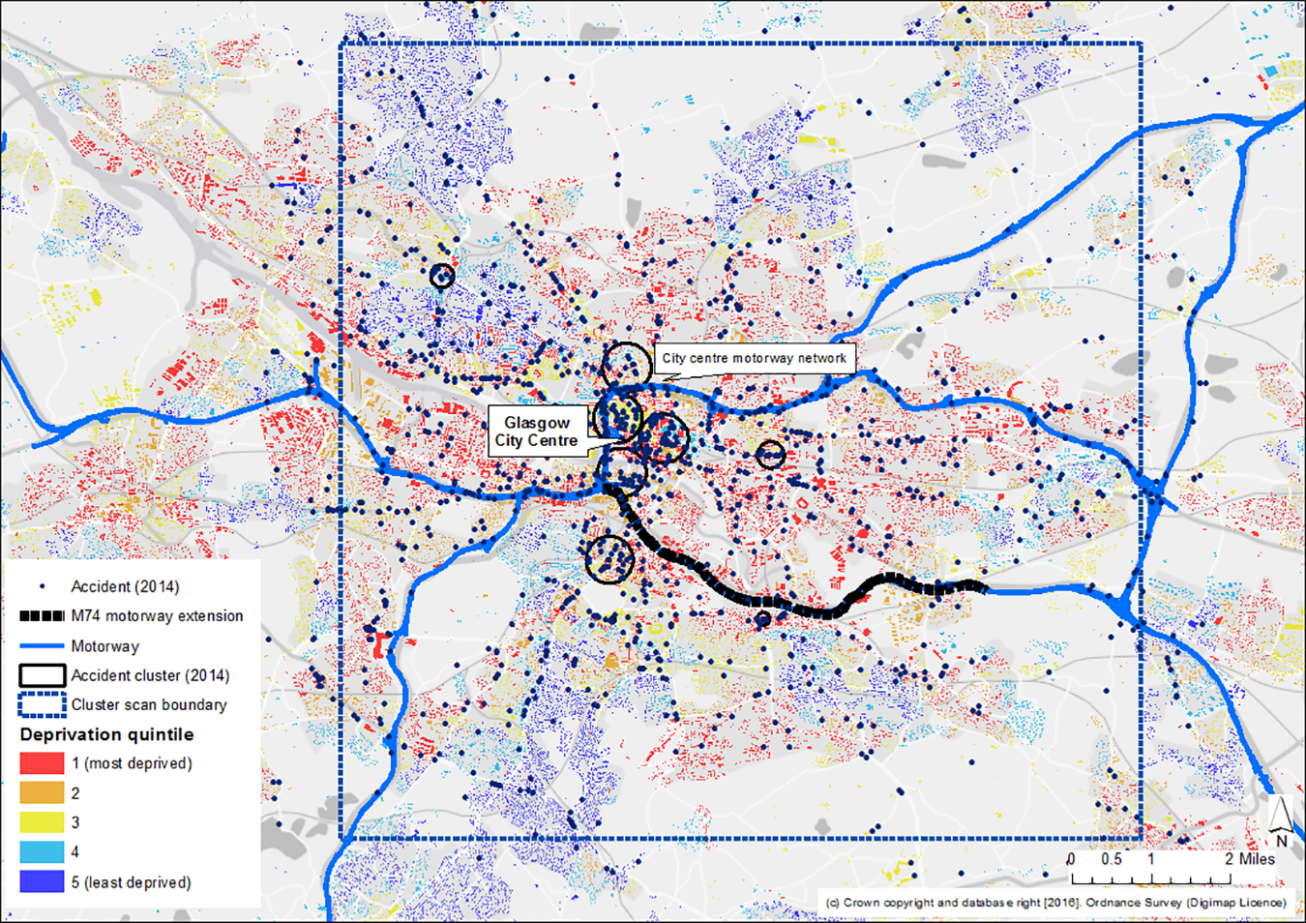
Accidents and spatial clusters of accidents in Glasgow, 2014. Note: Scottish Indices of Multiple Deprivation (SIMD) 2012 quintiles assigned to buildings within datazone administrative boundaries. Reprinted from Edina Digimap under a CC BY license, with permission from Ordnance Survey, original copyright 2016.

**Fig 4:**
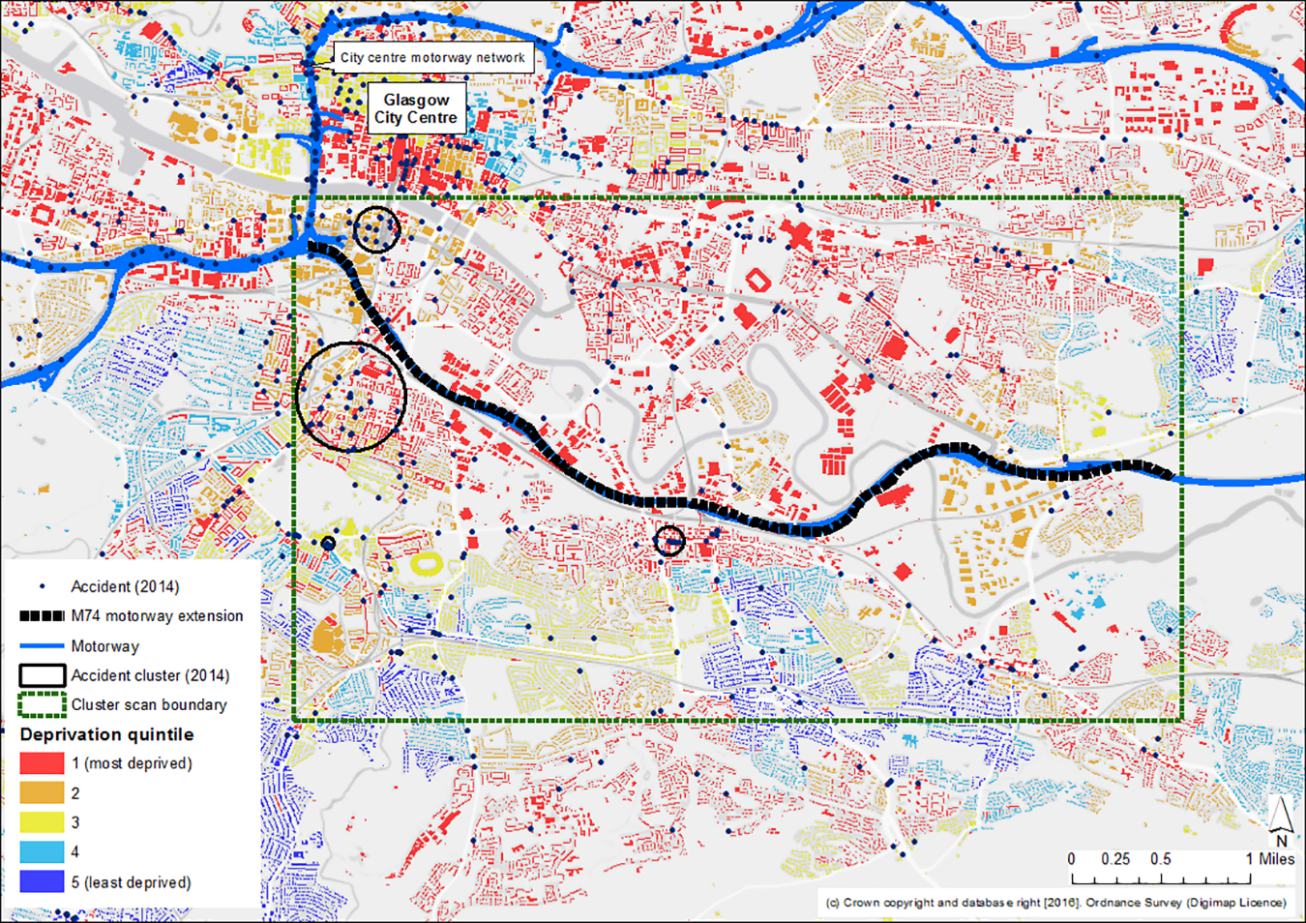
Accidents and spatial clusters of accidents in the area surrounding the M74 extension, 2014. Note: Scottish Indices of Multiple Deprivation (SIMD) 2012 quintiles assigned to buildings within datazone administrative boundaries. Reprinted from Edina Digimap under a CC BY license, with permission from Ordnance Survey, original copyright 2016.

Accident clusters were located in the city centre, on the motorway network and on other main non-motorway roads in the city. This pattern was consistent across the whole period of the study. As an example, Figs 3 and 4 illustrate the locations of accidents and clusters both city-wide and in the area surrounding the M74 extension for the most recent time period. They also portray the geography of socio-economic deprivation and corroborate Fig 2 in showing that most accidents, and most accident clusters, occurred in the more deprived areas of the city.

#### Spatiotemporal change of accident clusters

There was no significant change in the spatial location of clusters during the time period under study, either in the area surrounding the M74 motorway extension or city-wide. There was also no evidence of new cluster formation or loss of existing clusters during the study period (Table 1). This suggests no effect on the spatial location of accident clusters of either the construction or the opening of the M74 extension. S1 Fig. illustrates that there were no large changes in location of accident clusters surrounding the M74 extension between the earliest and latest time periods. During 2008, a borderline significant cluster was identified, cluster number 1 (Table 1), but this cluster was not identified in the later time-periods and is unlikely to be associated with the opening of the M74 extension. The three-year construction period began in mid-2008, and as this cluster was not sustained further during the full construction period it is unlikely to be associated with this.

**Table 1:** Spatial-temporal analysis of accident clusters in area surrounding the M74 motorway extension.

Cluster No.	X coordinate	Y coordinate	Cluster start date	Cluster end date	Likelihood ratio test	P value
1	262490	664190	01/01/2008	31/12/2008	10.1	0.06
2	258740	660940	01/01/2008	31/12/2008	8.4	0.33
3	258153	661617	01/01/2008	31/12/2008	7.3	0.76
6	260630	664480	01/01/2008	31/12/2008	6.7	0.95
4	264310	660390	01/01/2010	31/12/2010	7.0	0.88
7	260820	663300	01/01/2010	31/12/2011	6.2	1.00
5	264330	661500	01/01/2012	31/12/2014	6.9	0.90

Table contains the location of accident clusters that changed during the study period (2008 to 2014) including the dates on which the cluster was first and last detected. The likelihood ratio test provides evidence of the elevated risk of an accident cluster changing over time in that location.

## Discussion

### Main findings

The principal aims of our study were to describe the spatial pattering of accidents in Glasgow from 2008 to 2014 and to evaluate any impacts of the M74 motorway extension, opened in 2011, on the spatial location and socio-economic context of accident clusters. Throughout the study period, accidents and accident clusters were generally more likely to occur in the city centre and other main non-motorway roads in the city, and in areas classified as socio-economically deprived. We found no significant change in the spatial location or socio-economic context of accident clusters from 2008 to 2014, either in the area surrounding the M74 extension, or city-wide. We consequently infer no impact of the M74 motorway extension on these aspects of the epidemiology of road traffic accidents.

### Comparison with existing literature

We found no other studies that have evaluated the impact of new road infrastructure on accident clusters. This was recently highlighted by Knoflacher (2017) who found that the question “What are the effects of a new motorway on the traffic safety of a region or city?” had not been asked [17] until our own recent study examining the impact on the M74 extension on numbers of accidents [19]. Previous studies have, however, used cluster analysis to identify unsafe bus stops (based on the spatial clustering of pedestrian-vehicle accidents in Adelaide, Australia) [35] and areas of high risk of accidents to child pedestrians (in Santiago, Chile) [36]. The latter study highlighted that accident clusters were located in the more deprived areas of the city, echoing our findings. Previous studies have also confirmed that accidents are not randomly geographically distributed [6], and in a study of pedestrian and bicycle accidents resulting in an trauma admission in New York, spatial analysis highlighted that clusters tended to occur in downtown city areas [6]. A study in Lazio, Italy mapped severe accidents and identified clusters via visual inspection [37]. Although formal statistical analysis was not conducted, the authors found, as we did, that accident clusters were mainly located within the city centre and larger urban zone.

City centres and larger urban zones may produce more accidents as, typically, traffic travels on older road networks which were not built to support current traffic levels, and there may be increased opportunity for lapses in both driver and pedestrian concentration resulting in accidents. Factors such as improved traffic management, traffic calming measures and road safety initiatives have substantially decreased the overall incidence of accidents in the U.K. over the past decade [38]. However, our study has shown that for Glasgow the city centre continues to contain the majority of accidents and accident clusters.

### Strengths and limitations

Our study has strengths. The STATS19 database records all accidents, including their precise geo-coordinates. Although the STATS19 dataset relies upon accurate documentation by police officers [39], it is widely used in research [38–40] and regarded as fit for this purpose. Because the area we analysed lies within a single police administrative area, the reporting procedures are likely to have been consistent across this geographical area.

Previous studies have described methods for accident cluster detection, such as kernel density estimates, but have concluded that such methods are limited by their inability to determine the statistical significance of the resulting clusters [2]. A key strength of our study was that the analytical procedure we applied provided objective, robust detection of accident clusters, and spatiotemporal changes in these clusters, with accompanying statistical data. We were also able to conduct a sensitivity analysis which allowed for possible city-wide effects of a localised intervention to be examined, recognising that urban traffic functions as a system rather than in local silos.

There are some limitations in the reporting of all accidents within STATS19. Minor accidents not attended by the police are only ascertained if reported by drivers, and this may result in their under-representation in the dataset [41]. Similarly, accidents not resulting in a casualty will not have been included in the dataset. A further limitation is that we were not able to compare our findings for Glasgow with a comparator or control city, which limits the causal inference that may be derived from the findings. This is a limitation of many natural experimental studies focused on context-specific interventions [42]. In studies of this kind it can be difficult or impossible to find another city with sufficiently comparable size, climate, population structure, road layout and design, let alone one with a comparable intervention or, alternatively, the complete absence of any other interventions that could have affected the outcomes. However, it appears unlikely that even including such a hypothetical control city would have changed the main messages of our results, given that we were able to track accidents over time and in relation to the opening of the M74 extension in a relatively robust ‘before and after’ evaluation design.

We made considerable efforts to obtain suitable traffic count denominator data for the study area and time period, but this was not possible. Our analysis is therefore of counts of accidents, rather than rates. Arguably, however, traffic density is a component of a mechanism which produces accidents and the public health outcome of interest for the analysis was accident occurrence rather than risk.

The cluster detection software often does not detect clusters close to borders [34], but by running sensitivity analysis we were able to extend the border to ensure that clusters were not missed. In the final analyses we used a smaller defined boundary which surrounded the city region of Glasgow and ensured that accidents occurring on a largely urban road network were included.

## Conclusions

We found no impact of a new section of urban motorway on the spatial distribution or socio-economic context of road traffic accidents. This adds to the existing evidence that the M74 motorway extension had little impact on accidents in the city [19]. Spatial and cluster detection of accidents is a valuable procedure that can help policymakers and planners understand where most accidents occur and the populations they mainly affect. We have identified clusters of accidents in Glasgow which are located primarily in the most deprived areas of the city, and have remained spatially static for a considerable time period. The availability of precise accident data and spatial analytical software enables road safety professionals to target these specific areas, and our findings illustrate how local area analysis could be used to identify and target accident clusters in other cities around the world.

## Supporting information

S1 Fig Accidents and spatial clusters of accidents in the area surrounding the M74 extension, 2008. Note: Scottish Indices of Multiple Deprivation (SIMD) 2012 quintiles assigned to buildings within datazone administrative boundaries. Reprinted from Edina Digimap under a CC BY license, with permission from Ordnance Survey, original copyright 2016.
(TIF)

S1 Table Boundary coordinates for SaTScan spatial cluster sensitivity analysis. (DOCX)

S2 Table Count of all accidents by year and deprivation quintile. (DOCX)

S3 Table Count of clustered accidents by year and deprivation quintile. (DOCX)

S1 File Documented permission from Ordnance Survey for publication of Edina Digimap under a CC BY licence. (TXT)
