# Supplementary figures and images for: Effect of a new motorway on social-spatial patterning of road traffic accidents: A retrospective longitudinal natural experimental study
Source: PLoS One. 2017 Sep 7;12(9):e0184047. doi: 10.1371/journal.pone.0184047 (PMC5589166; doi:10.1371/journal.pone.0184047)

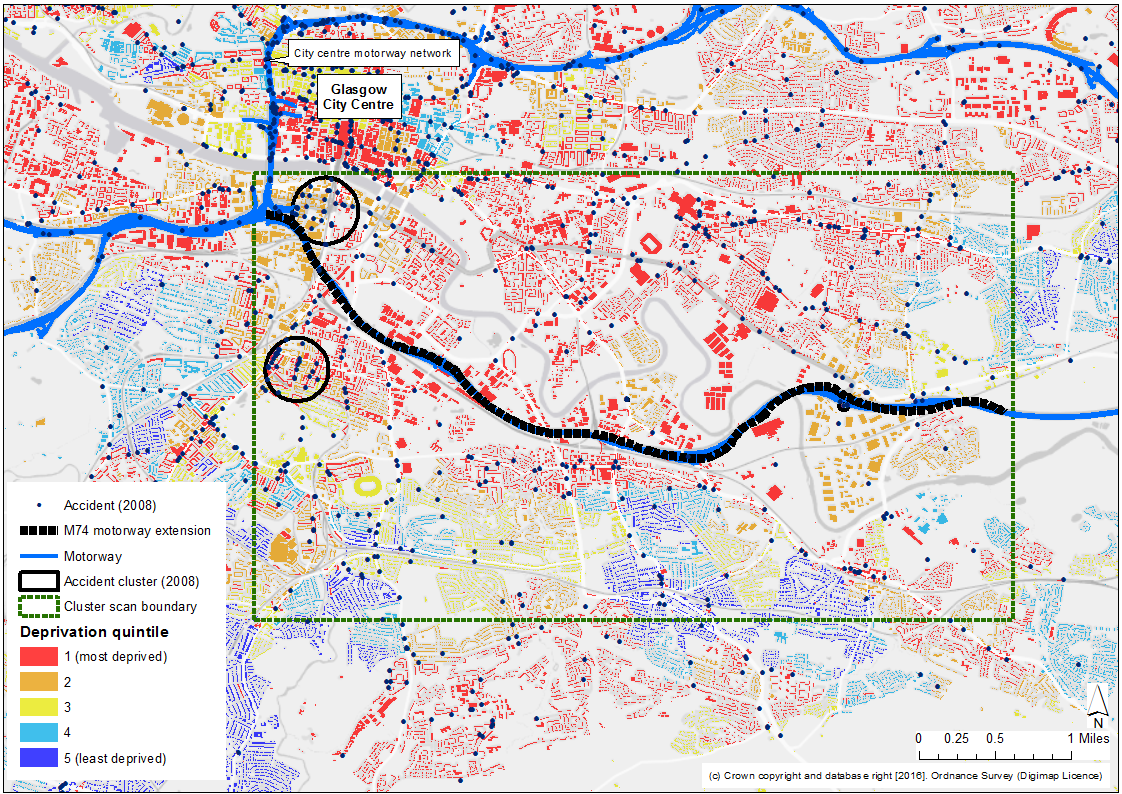

Supplement: S1 Fig — Note: Scottish Indices of Multiple Deprivation (SIMD) 2012 quintiles assigned to buildings within datazone administrative boundaries. Reprinted from Edina Digimap under a CC BY license, with permission from Ordnance Survey, original copyright 2016. (TIF) [file pone.0184047.s001.tif]
