# Supplementary material for: Effect of a new motorway on social-spatial patterning of road traffic accidents: A retrospective longitudinal natural experimental study
Source: PLoS One. 2017 Sep 7;12(9):e0184047. doi: 10.1371/journal.pone.0184047 (PMC5589166; doi:10.1371/journal.pone.0184047)
Supplement: S1 Table — (DOCX) [file pone.0184047.s002.docx]

**S1 Table. Boundary coordinates for SaTScan spatial cluster sensitivity analysis*.***

| **Boundary** | **Y coordinates** | **X coordinates** | **Area within boundary (km^2^)** |
| --- | --- | --- | --- |
| Small | 660680, 671134 | 254833, 266330 | 120 |
| Medium | 657173, 673429 | 252594, 268935 | 266 |
| Large | 656376, 675823 | 251021, 270335 | 376 |
| Local boundary surrounding M74 extension | 664793, 660043 | 257808, 265851 | 31 |
