# Supplementary material for: Effect of a new motorway on social-spatial patterning of road traffic accidents: A retrospective longitudinal natural experimental study
Source: PLoS One. 2017 Sep 7;12(9):e0184047. doi: 10.1371/journal.pone.0184047 (PMC5589166; doi:10.1371/journal.pone.0184047)
Supplement: S2 Table — (DOCX) [file pone.0184047.s003.docx]

**S2 Table. Count of all accidents by year and deprivation quintile.**

|  | | Year | | | | | | |
| --- | --- | --- | --- | --- | --- | --- | --- | --- |
|  |  | 2008 | 2009 | 2010 | 2011 | 2012 | 2013 | 2014 |
| Deprivation quintile | 1 (most deprived) | 1417 | 1240 | 1128 | 1047 | 989 | 888 | 973 |
|  | 2 | 919 | 871 | 774 | 744 | 704 | 608 | 646 |
|  | 3 | 722 | 710 | 620 | 661 | 672 | 586 | 559 |
|  | 4 | 537 | 521 | 501 | 491 | 439 | 406 | 443 |
|  | 5 (least deprived) | 290 | 278 | 260 | 291 | 257 | 245 | 287 |
