# Supplementary material for: Effect of a new motorway on social-spatial patterning of road traffic accidents: A retrospective longitudinal natural experimental study
Source: PLoS One. 2017 Sep 7;12(9):e0184047. doi: 10.1371/journal.pone.0184047 (PMC5589166; doi:10.1371/journal.pone.0184047)
Supplement: S3 Table — (DOCX) [file pone.0184047.s004.docx]

**S3 Table. Count of clustered accidents by year and deprivation quintile.**

|  | | Year | | | | | | |
| --- | --- | --- | --- | --- | --- | --- | --- | --- |
|  |  | 2008 | 2009 | 2010 | 2011 | 2012 | 2013 | 2014 |
| Deprivation quintile | 1 (most deprived) | 174 | 99 | 108 | 114 | 77 | 88 | 90 |
|  | 2 | 133 | 94 | 76 | 103 | 83 | 65 | 87 |
|  | 3 | 137 | 134 | 117 | 71 | 105 | 76 | 84 |
|  | 4 | 26 | 42 | 26 | 48 | 33 | 31 | 30 |
|  | 5 (least deprived) | 1 | 11 | 0 | 1 | 2 | 3 | 3 |
